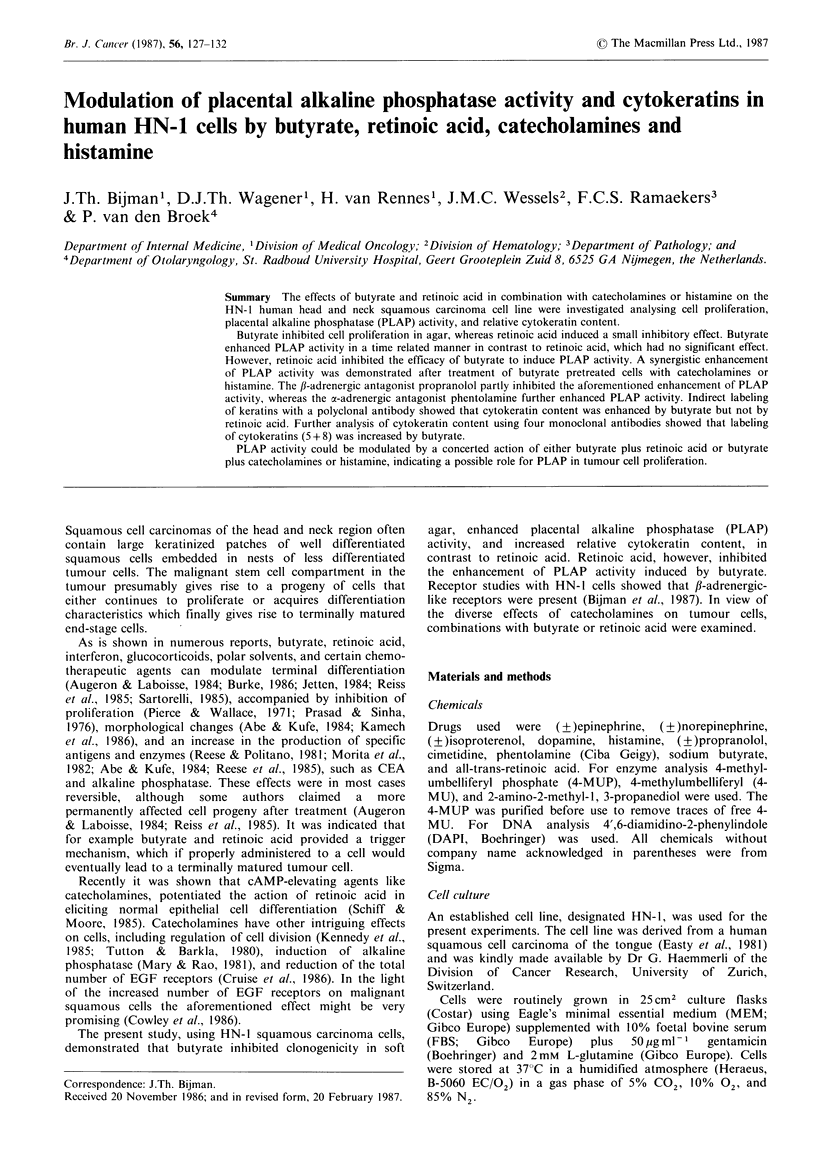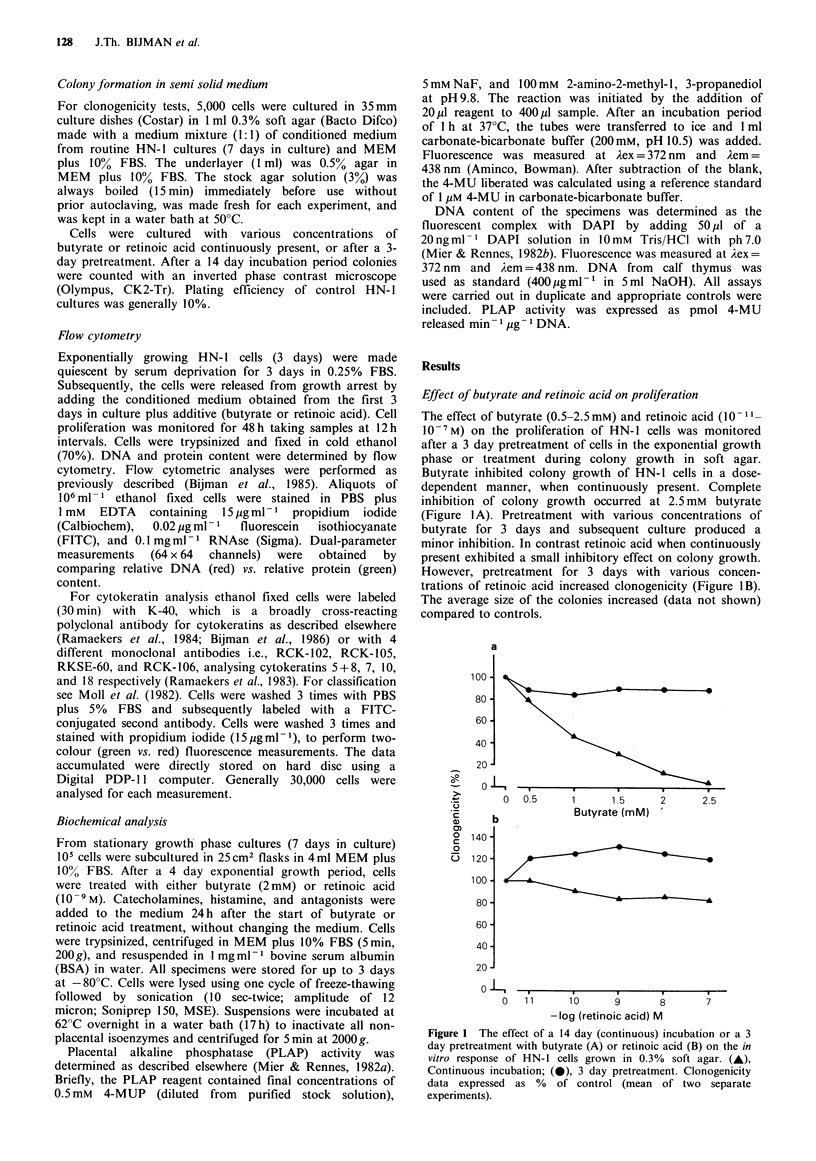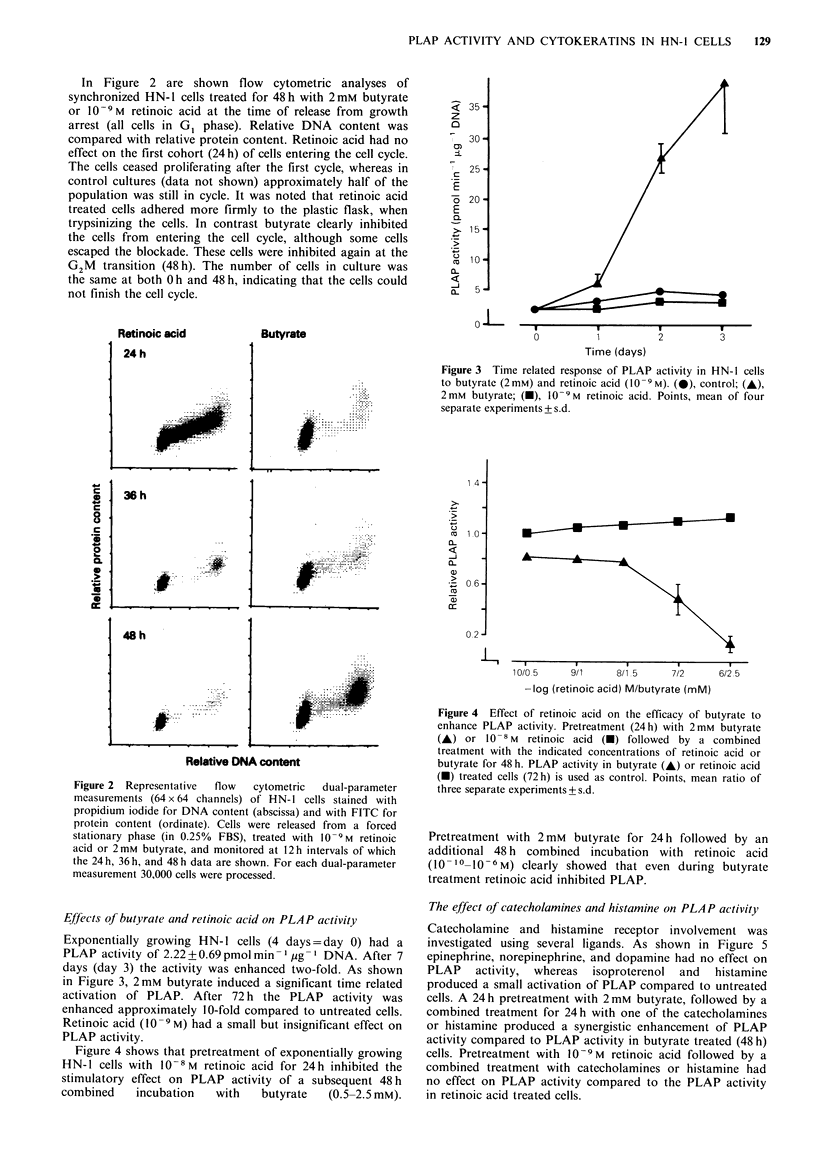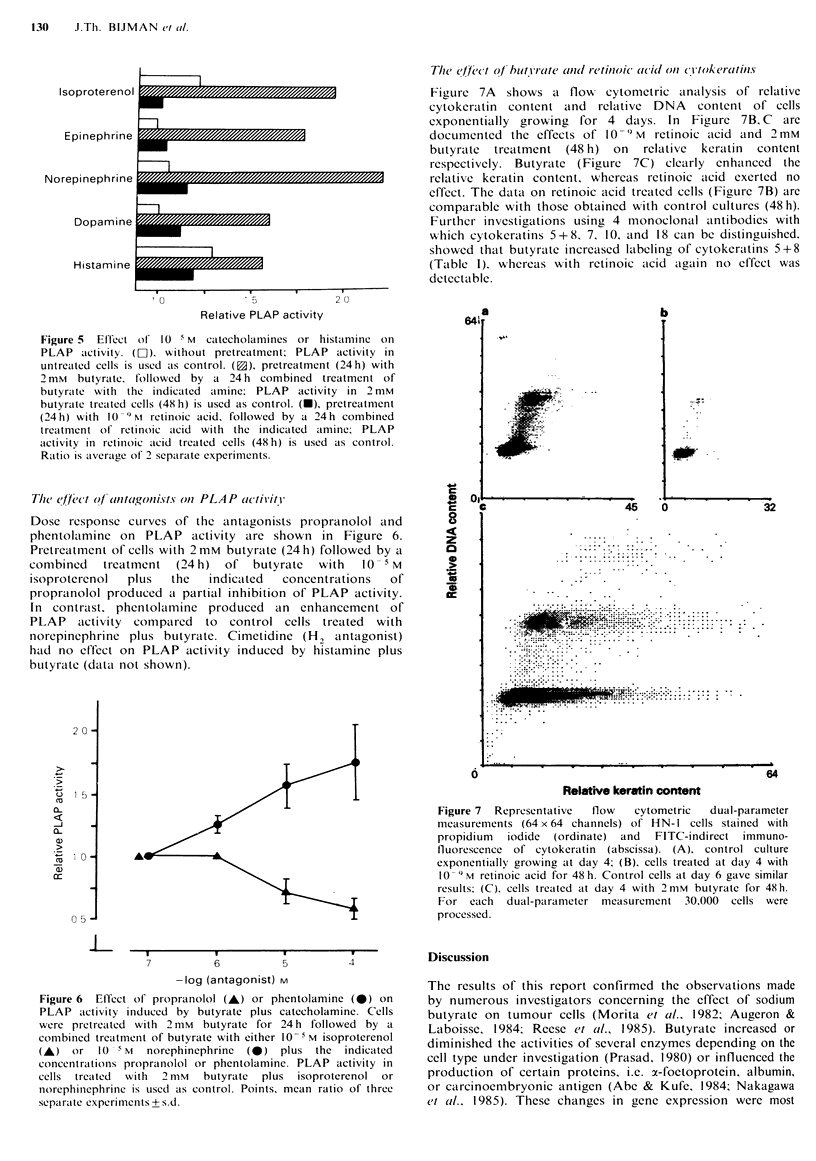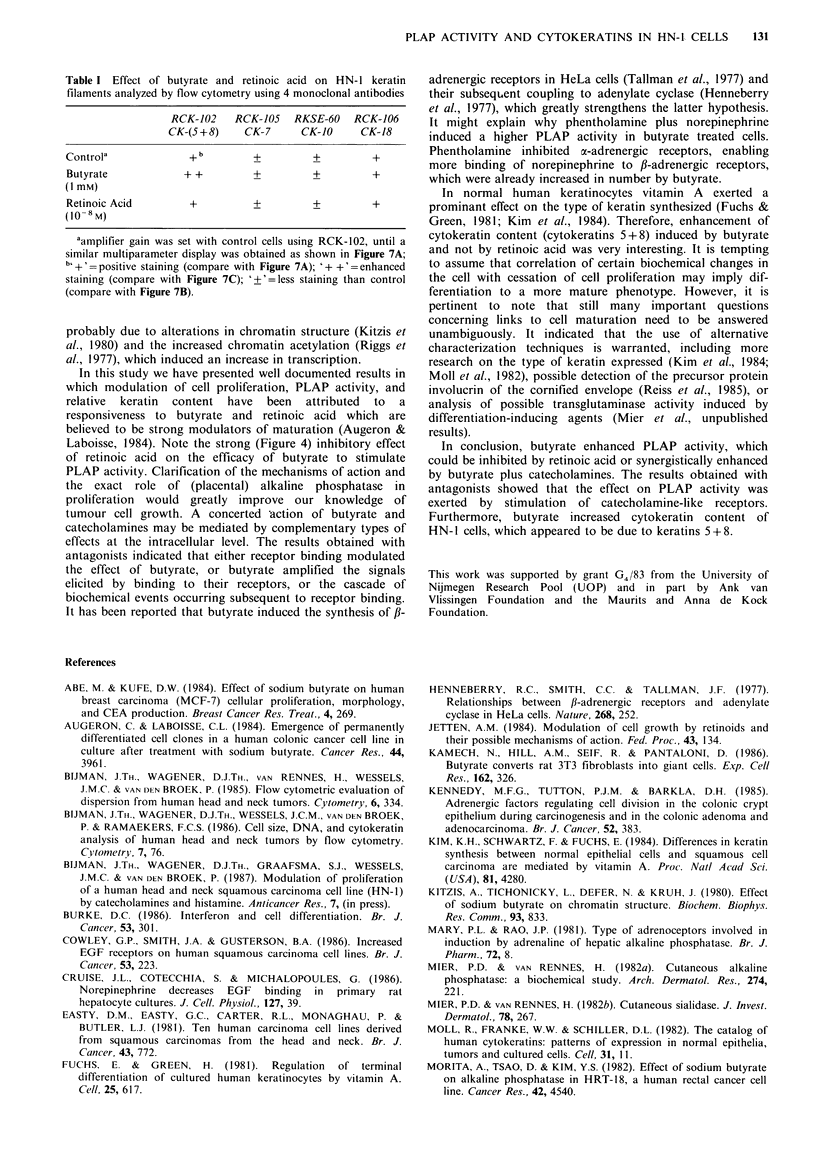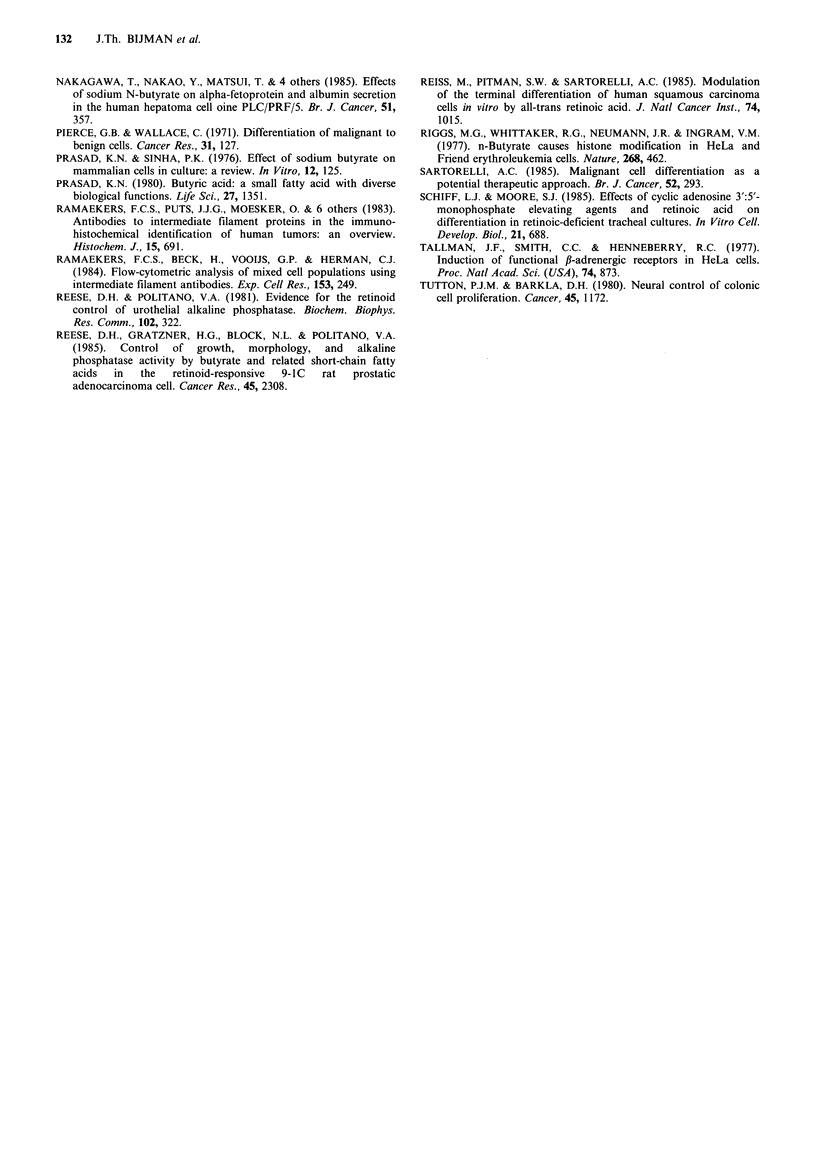# Modulation of placental alkaline phosphatase activity and cytokeratins in human HN-1 cells by butyrate, retinoic acid, catecholamines and histamine.

**DOI:** 10.1038/bjc.1987.169

**Published:** 1987-08

**Authors:** J. T. Bijman, D. J. Wagener, H. van Rennes, J. M. Wessels, F. C. Ramaekers, P. van den Broek

**Affiliations:** Division of Medical Oncology, St. Radboud University Hospital, Nijmegen, The Netherlands.

## Abstract

The effects of butyrate and retinoic acid in combination with catecholamines or histamine on the HN-1 human head and neck squamous carcinoma cell line were investigated analysing cell proliferation, placental alkaline phosphatase (PLAP) activity, and relative cytokeratin content. Butyrate inhibited cell proliferation in agar, whereas retinoic acid induced a small inhibitory effect. Butyrate enhanced PLAP activity in a time related manner in contrast to retinoic acid, which had no significant effect. However, retinoic acid inhibited the efficacy of butyrate to induce PLAP activity. A synergistic enhancement of PLAP activity was demonstrated after treatment of butyrate pretreated cells with catecholamines or histamine. The beta-adrenergic antagonist propranolol partly inhibited the aforementioned enhancement of PLAP activity, whereas the alpha-adrenergic antagonist phentolamine further enhanced PLAP activity. Indirect labeling of keratins with a polyclonal antibody showed that cytokeratin content was enhanced by butyrate but not by retinoic acid. Further analysis of cytokeratin content using four monoclonal antibodies showed that labeling of cytokeratins (5 + 8) was increased by butyrate. PLAP activity could be modulated by a concerted action of either butyrate plus retinoic acid or butyrate plus catecholamines or histamine, indicating a possible role for PLAP in tumour cell proliferation.


Br. J. Cancer (1987), 56, 127–132 © The Macmillan Press Ltd., 1987

### Modulation of placental alkaline phosphatase activity and cytokeratins in human HN-1 cells by butyrate, retinoic acid, catecholamines and histamine

J.Th. Bijman[1], D.J.Th. Wagener[1], H. van Rennes[1], J.M.C. Wessels[2], F.C.S. Ramaekers[3] & P. van den Broek[4]

*Department of Internal Medicine, [1]Division of Medical Oncology; [2]Division of Hematology; [3]Department of Pathology; and [4]Department of Otolaryngology, St. Radboud University Hospital, Geert Grooteplein Zuid 8, 6525 GA Nijmegen, the Netherlands.*

**Summary** The effects of butyrate and retinoic acid in combination with catecholamines or histamine on the HN-1 human head and neck squamous carcinoma cell line were investigated analysing cell proliferation, placental alkaline phosphatase (PLAP) activity, and relative cytokeratin content.

Butyrate inhibited cell proliferation in agar, whereas retinoic acid induced a small inhibitory effect. Butyrate enhanced PLAP activity in a time related manner in contrast to retinoic acid, which had no significant effect. However, retinoic acid inhibited the efficacy of butyrate to induce PLAP activity. A synergistic enhancement of PLAP activity was demonstrated after treatment of butyrate pretreated cells with catecholamines or histamine. The $\beta$-adrenergic antagonist propranolol partly inhibited the aforementioned enhancement of PLAP activity, whereas the $\alpha$-adrenergic antagonist phentolamine further enhanced PLAP activity. Indirect labeling of keratins with a polyclonal antibody showed that cytokeratin content was enhanced by butyrate but not by retinoic acid. Further analysis of cytokeratin content using four monoclonal antibodies showed that labeling of cytokeratins (5+8) was increased by butyrate.

PLAP activity could be modulated by a concerted action of either butyrate plus retinoic acid or butyrate plus catecholamines or histamine, indicating a possible role for PLAP in tumour cell proliferation.

Squamous cell carcinomas of the head and neck region often contain large keratinized patches of well differentiated squamous cells embedded in nests of less differentiated tumour cells. The malignant stem cell compartment in the tumour presumably gives rise to a progeny of cells that either continues to proliferate or acquires differentiation characteristics which finally gives rise to terminally matured end-stage cells.

As is shown in numerous reports, butyrate, retinoic acid, interferon, glucocorticoids, polar solvents, and certain chemotherapeutic agents can modulate terminal differentiation (Augeron & Laboisse, 1984; Burke, 1986; Jetten, 1984; Reiss *et al.*, 1985; Sartorelli, 1985), accompanied by inhibition of proliferation (Pierce & Wallace, 1971; Prasad & Sinha, 1976), morphological changes (Abe & Kufe, 1984; Kamech *et al.*, 1986), and an increase in the production of specific antigens and enzymes (Reese & Politano, 1981; Morita *et al.*, 1982; Abe & Kufe, 1984; Reese *et al.*, 1985), such as CEA and alkaline phosphatase. These effects were in most cases reversible, although some authors claimed a more permanently affected cell progeny after treatment (Augeron & Laboisse, 1984; Reiss *et al.*, 1985). It was indicated that for example butyrate and retinoic acid provided a trigger mechanism, which if properly administered to a cell would eventually lead to a terminally matured tumour cell.

Recently it was shown that cAMP-elevating agents like catecholamines, potentiated the action of retinoic acid in eliciting normal epithelial cell differentiation (Schiff & Moore, 1985). Catecholamines have other intriguing effects on cells, including regulation of cell division (Kennedy *et al.*, 1985; Tutton & Barkla, 1980), induction of alkaline phosphatase (Mary & Rao, 1981), and reduction of the total number of EGF receptors (Cruise *et al.*, 1986). In the light of the increased number of EGF receptors on malignant squamous cells the aforementioned effect might be very promising (Cowley *et al.*, 1986).

The present study, using HN-1 squamous carcinoma cells, demonstrated that butyrate inhibited clonogenicity in soft agar, enhanced placental alkaline phosphatase (PLAP) activity, and increased relative cytokeratin content, in contrast to retinoic acid. Retinoic acid, however, inhibited the enhancement of PLAP activity induced by butyrate. Receptor studies with HN-1 cells showed that $\beta$-adrenergic-like receptors were present (Bijman *et al.*, 1987). In view of the diverse effects of catecholamines on tumour cells, combinations with butyrate or retinoic acid were examined.

### Materials and methods

### *Chemicals*

Drugs used were (±)epinephrine, (±)norepinephrine, (±)isoproterenol, dopamine, histamine, (±)propranolol, cimetidine, phentolamine (Ciba Geigy), sodium butyrate, and all-trans-retinoic acid. For enzyme analysis 4-methylumbelliferyl phosphate (4-MUP), 4-methylumbelliferyl (4-MU), and 2-amino-2-methyl-1, 3-propanediol were used. The 4-MUP was purified before use to remove traces of free 4-MU. For DNA analysis 4′,6-diamidino-2-phenylindole (DAPI, Boehringer) was used. All chemicals without company name acknowledged in parentheses were from Sigma.

### *Cell culture*

An established cell line, designated HN-1, was used for the present experiments. The cell line was derived from a human squamous cell carcinoma of the tongue (Easty *et al.*, 1981) and was kindly made available by Dr G. Haemmerli of the Division of Cancer Research, University of Zurich, Switzerland.

Cells were routinely grown in 25 cm$^2$ culture flasks (Costar) using Eagle's minimal essential medium (MEM; Gibco Europe) supplemented with 10% foetal bovine serum (FBS; Gibco Europe) plus 50 $\mu$g ml$^{-1}$ gentamicin (Boehringer) and 2 mM L-glutamine (Gibco Europe). Cells were stored at 37°C in a humidified atmosphere (Heraeus, B-5060 EC/$O_2$) in a gas phase of 5% $CO_2$, 10% $O_2$, and 85% $N_2$.

Correspondence: J.Th. Bijman.
Received 20 November 1986; and in revised form, 20 February 1987.

*Colony formation in semi solid medium*

For clonogenicity tests, 5,000 cells were cultured in 35 mm culture dishes (Costar) in 1 ml 0.3% soft agar (Bacto Difco) made with a medium mixture (1:1) of conditioned medium from routine HN-1 cultures (7 days in culture) and MEM plus 10% FBS. The underlayer (1 ml) was 0.5% agar in MEM plus 10% FBS. The stock agar solution (3%) was always boiled (15 min) immediately before use without prior autoclaving, was made fresh for each experiment, and was kept in a water bath at 50°C.

Cells were cultured with various concentrations of butyrate or retinoic acid continuously present, or after a 3-day pretreatment. After a 14 day incubation period colonies were counted with an inverted phase contrast microscope (Olympus, CK2-Tr). Plating efficiency of control HN-1 cultures was generally 10%.

*Flow cytometry*

Exponentially growing HN-1 cells (3 days) were made quiescent by serum deprivation for 3 days in 0.25% FBS. Subsequently, the cells were released from growth arrest by adding the conditioned medium obtained from the first 3 days in culture plus additive (butyrate or retinoic acid). Cell proliferation was monitored for 48 h taking samples at 12 h intervals. Cells were trypsinized and fixed in cold ethanol (70%). DNA and protein content were determined by flow cytometry. Flow cytometric analyses were performed as previously described (Bijman *et al.*, 1985). Aliquots of $10^6$ ml$^{-1}$ ethanol fixed cells were stained in PBS plus 1 mM EDTA containing 15 μg ml$^{-1}$ propidium iodide (Calbiochem), 0.02 μg ml$^{-1}$ fluorescein isothiocyanate (FITC), and 0.1 mg ml$^{-1}$ RNAse (Sigma). Dual-parameter measurements (64 x 64 channels) were obtained by comparing relative DNA (red) *vs.* relative protein (green) content.

For cytokeratin analysis ethanol fixed cells were labeled (30 min) with K-40, which is a broadly cross-reacting polyclonal antibody for cytokeratins as described elsewhere (Ramaekers *et al.*, 1984; Bijman *et al.*, 1986) or with 4 different monoclonal antibodies i.e., RCK-102, RCK-105, RKSE-60, and RCK-106, analysing cytokeratins 5+8, 7, 10, and 18 respectively (Ramaekers *et al.*, 1983). For classification see Moll *et al.* (1982). Cells were washed 3 times with PBS plus 5% FBS and subsequently labeled with a FITC-conjugated second antibody. Cells were washed 3 times and stained with propidium iodide (15 μg ml$^{-1}$), to perform two-colour (green *vs.* red) fluorescence measurements. The data accumulated were directly stored on hard disc using a Digital PDP-11 computer. Generally 30,000 cells were analysed for each measurement.

*Biochemical analysis*

From stationary growth phase cultures (7 days in culture) $10^5$ cells were subcultured in 25 cm$^2$ flasks in 4 ml MEM plus 10% FBS. After a 4 day exponential growth period, cells were treated with either butyrate (2 mM) or retinoic acid ($10^{-9}$ M). Catecholamines, histamine, and antagonists were added to the medium 24 h after the start of butyrate or retinoic acid treatment, without changing the medium. Cells were trypsinized, centrifuged in MEM plus 10% FBS (5 min, 200 *g*), and resuspended in 1 mg ml$^{-1}$ bovine serum albumin (BSA) in water. All specimens were stored for up to 3 days at −80°C. Cells were lysed using one cycle of freeze-thawing followed by sonication (10 sec-twice; amplitude of 12 micron; Soniprep 150, MSE). Suspensions were incubated at 62°C overnight in a water bath (17 h) to inactivate all non-placental isoenzymes and centrifuged for 5 min at 2000 *g*.

Placental alkaline phosphatase (PLAP) activity was determined as described elsewhere (Mier & Rennes, 1982*a*). Briefly, the PLAP reagent contained final concentrations of 0.5 mM 4-MUP (diluted from purified stock solution), 5 mM NaF, and 100 mM 2-amino-2-methyl-1, 3-propanediol at pH 9.8. The reaction was initiated by the addition of 20 μl reagent to 400 μl sample. After an incubation period of 1 h at 37°C, the tubes were transferred to ice and 1 ml carbonate-bicarbonate buffer (200 mM, pH 10.5) was added. Fluorescence was measured at λex = 372 nm and λem = 438 nm (Aminco, Bowman). After subtraction of the blank, the 4-MU liberated was calculated using a reference standard of 1 μM 4-MU in carbonate-bicarbonate buffer.

DNA content of the specimens was determined as the fluorescent complex with DAPI by adding 50 μl of a 20 ng ml$^{-1}$ DAPI solution in 10 mM Tris/HCl with ph 7.0 (Mier & Rennes, 1982*b*). Fluorescence was measured at λex = 372 nm and λem = 438 nm. DNA from calf thymus was used as standard (400 μg ml$^{-1}$ in 5 ml NaOH). All assays were carried out in duplicate and appropriate controls were included. PLAP activity was expressed as pmol 4-MU released min$^{-1}$ μg$^{-1}$ DNA.

### Results

*Effect of butyrate and retinoic acid on proliferation*

The effect of butyrate (0.5–2.5 mM) and retinoic acid ($10^{-11}$–$10^{-7}$ M) on the proliferation of HN-1 cells was monitored after a 3 day pretreatment of cells in the exponential growth phase or treatment during colony growth in soft agar. Butyrate inhibited colony growth of HN-1 cells in a dose-dependent manner, when continuously present. Complete inhibition of colony growth occurred at 2.5 mM butyrate (Figure 1A). Pretreatment with various concentrations of butyrate for 3 days and subsequent culture produced a minor inhibition. In contrast retinoic acid when continuously present exhibited a small inhibitory effect on colony growth. However, pretreatment for 3 days with various concentrations of retinoic acid increased clonogenicity (Figure 1B). The average size of the colonies increased (data not shown) compared to controls.

**Figure 1** The effect of a 14 day (continuous) incubation or a 3 day pretreatment with butyrate (A) or retinoic acid (B) on the *in vitro* response of HN-1 cells grown in 0.3% soft agar. (▲), Continuous incubation; (●), 3 day pretreatment. Clonogenicity data expressed as % of control (mean of two separate experiments).

In Figure 2 are shown flow cytometric analyses of synchronized HN-1 cells treated for 48 h with 2 mM butyrate or $10^{-9}$ M retinoic acid at the time of release from growth arrest (all cells in $G_1$ phase). Relative DNA content was compared with relative protein content. Retinoic acid had no effect on the first cohort (24 h) of cells entering the cell cycle. The cells ceased proliferating after the first cycle, whereas in control cultures (data not shown) approximately half of the population was still in cycle. It was noted that retinoic acid treated cells adhered more firmly to the plastic flask, when trypsinizing the cells. In contrast butyrate clearly inhibited the cells from entering the cell cycle, although some cells escaped the blockade. These cells were inhibited again at the $G_2M$ transition (48 h). The number of cells in culture was the same at both 0 h and 48 h, indicating that the cells could not finish the cell cycle.

**Figure 2** Representative flow cytometric dual-parameter measurements (64 × 64 channels) of HN-1 cells stained with propidium iodide for DNA content (abscissa) and with FITC for protein content (ordinate). Cells were released from a forced stationary phase (in 0.25% FBS), treated with $10^{-9}$ M retinoic acid or 2 mM butyrate, and monitored at 12 h intervals of which the 24 h, 36 h, and 48 h data are shown. For each dual-parameter measurement 30,000 cells were processed.

*Effects of butyrate and retinoic acid on PLAP activity*

Exponentially growing HN-1 cells (4 days = day 0) had a PLAP activity of 2.22 ± 0.69 pmol min$^{-1}$ µg$^{-1}$ DNA. After 7 days (day 3) the activity was enhanced two-fold. As shown in Figure 3, 2 mM butyrate induced a significant time related activation of PLAP. After 72 h the PLAP activity was enhanced approximately 10-fold compared to untreated cells. Retinoic acid ($10^{-9}$ M) had a small but insignificant effect on PLAP activity.

Figure 4 shows that pretreatment of exponentially growing HN-1 cells with $10^{-8}$ M retinoic acid for 24 h inhibited the stimulatory effect on PLAP activity of a subsequent 48 h combined incubation with butyrate (0.5–2.5 mM).

**Figure 3** Time related response of PLAP activity in HN-1 cells to butyrate (2 mM) and retinoic acid ($10^{-9}$ M). (●), control; (▲), 2 mM butyrate; (■), $10^{-9}$ M retinoic acid. Points, mean of four separate experiments ± s.d.

**Figure 4** Effect of retinoic acid on the efficacy of butyrate to enhance PLAP activity. Pretreatment (24 h) with 2 mM butyrate (▲) or $10^{-8}$ M retinoic acid (■) followed by a combined treatment with the indicated concentrations of retinoic acid or butyrate for 48 h. PLAP activity in butyrate (▲) or retinoic acid (■) treated cells (72 h) is used as control. Points, mean ratio of three separate experiments ± s.d.

Pretreatment with 2 mM butyrate for 24 h followed by an additional 48 h combined incubation with retinoic acid ($10^{-10}$–$10^{-6}$ M) clearly showed that even during butyrate treatment retinoic acid inhibited PLAP.

*The effect of catecholamines and histamine on PLAP activity*

Catecholamine and histamine receptor involvement was investigated using several ligands. As shown in Figure 5 epinephrine, norepinephrine, and dopamine had no effect on PLAP activity, whereas isoproterenol and histamine produced a small activation of PLAP compared to untreated cells. A 24 h pretreatment with 2 mM butyrate, followed by a combined treatment for 24 h with one of the catecholamines or histamine produced a synergistic enhancement of PLAP activity compared to PLAP activity in butyrate treated (48 h) cells. Pretreatment with $10^{-9}$ M retinoic acid followed by a combined treatment with catecholamines or histamine had no effect on PLAP activity compared to the PLAP activity in retinoic acid treated cells.

**Figure 5** Effect of $10^{-5}$ M catecholamines or histamine on PLAP activity. (□), without pretreatment; PLAP activity in untreated cells is used as control. (▨), pretreatment (24 h) with 2 mM butyrate, followed by a 24 h combined treatment of butyrate with the indicated amine; PLAP activity in 2 mM butyrate treated cells (48 h) is used as control. (■), pretreatment (24 h) with $10^{-9}$ M retinoic acid, followed by a 24 h combined treatment of retinoic acid with the indicated amine; PLAP activity in retinoic acid treated cells (48 h) is used as control. Ratio is average of 2 separate experiments.

*The effect of antagonists on PLAP activity*

Dose response curves of the antagonists propranolol and phentolamine on PLAP activity are shown in Figure 6. Pretreatment of cells with 2 mM butyrate (24 h) followed by a combined treatment (24 h) of butyrate with $10^{-5}$ M isoproterenol plus the indicated concentrations of propranolol produced a partial inhibition of PLAP activity. In contrast, phentolamine produced an enhancement of PLAP activity compared to control cells treated with norepinephrine plus butyrate. Cimetidine ($H_2$ antagonist) had no effect on PLAP activity induced by histamine plus butyrate (data not shown).

**Figure 6** Effect of propranolol (▲) or phentolamine (●) on PLAP activity induced by butyrate plus catecholamine. Cells were pretreated with 2 mM butyrate for 24 h followed by a combined treatment of butyrate with either $10^{-5}$ M isoproterenol (▲) or $10^{-5}$ M norephinephrine (●) plus the indicated concentrations propranolol or phentolamine. PLAP activity in cells treated with 2 mM butyrate plus isoproterenol or norephinephrine is used as control. Points, mean ratio of three separate experiments ± s.d.

*The effect of butyrate and retinoic acid on cytokeratins*

Figure 7A shows a flow cytometric analysis of relative cytokeratin content and relative DNA content of cells exponentially growing for 4 days. In Figure 7B, C are documented the effects of $10^{-9}$ M retinoic acid and 2 mM butyrate treatment (48 h) on relative keratin content respectively. Butyrate (Figure 7C) clearly enhanced the relative keratin content, whereas retinoic acid exerted no effect. The data on retinoic acid treated cells (Figure 7B) are comparable with those obtained with control cultures (48 h). Further investigations using 4 monoclonal antibodies with which cytokeratins 5 + 8, 7, 10, and 18 can be distinguished, showed that butyrate increased labeling of cytokeratins 5 + 8 (Table I), whereas with retinoic acid again no effect was detectable.

**Figure 7** Representative flow cytometric dual-parameter measurements (64 × 64 channels) of HN-1 cells stained with propidium iodide (ordinate) and FITC-indirect immunofluorescence of cytokeratin (abscissa). (A), control culture exponentially growing at day 4; (B), cells treated at day 4 with $10^{-9}$ M retinoic acid for 48 h. Control cells at day 6 gave similar results; (C), cells treated at day 4 with 2 mM butyrate for 48 h. For each dual-parameter measurement 30,000 cells were processed.

### Discussion

The results of this report confirmed the observations made by numerous investigators concerning the effect of sodium butyrate on tumour cells (Morita *et al.*, 1982; Augeron & Laboisse, 1984; Reese *et al.*, 1985). Butyrate increased or diminished the activities of several enzymes depending on the cell type under investigation (Prasad, 1980) or influenced the production of certain proteins, i.e. α-foetoprotein, albumin, or carcinoembryonic antigen (Abe & Kufe, 1984; Nakagawa *et al.*, 1985). These changes in gene expression were most

**Table I** Effect of butyrate and retinoic acid on HN-1 keratin filaments analyzed by flow cytometry using 4 monoclonal antibodies

| | *RCK-102* *CK-(5+8)* | *RCK-105* *CK-7* | *RKSE-60* *CK-10* | *RCK-106* *CK-18* |
|---|---|---|---|---|
| Control[a] | +[b] | ± | ± | + |
| Butyrate (1 mM) | ++ | ± | ± | + |
| Retinoic Acid ($10^{-8}$ M) | + | ± | ± | + |

[a]amplifier gain was set with control cells using RCK-102, until a similar multiparameter display was obtained as shown in **Figure 7A**; [b]'+' = positive staining (compare with **Figure 7A**); '++' = enhanced staining (compare with **Figure 7C**); '±' = less staining than control (compare with **Figure 7B**).

probably due to alterations in chromatin structure (Kitzis *et al.*, 1980) and the increased chromatin acetylation (Riggs *et al.*, 1977), which induced an increase in transcription.

In this study we have presented well documented results in which modulation of cell proliferation, PLAP activity, and relative keratin content have been attributed to a responsiveness to butyrate and retinoic acid which are believed to be strong modulators of maturation (Augeron & Laboisse, 1984). Note the strong (Figure 4) inhibitory effect of retinoic acid on the efficacy of butyrate to stimulate PLAP activity. Clarification of the mechanisms of action and the exact role of (placental) alkaline phosphatase in proliferation would greatly improve our knowledge of tumour cell growth. A concerted action of butyrate and catecholamines may be mediated by complementary types of effects at the intracellular level. The results obtained with antagonists indicated that either receptor binding modulated the effect of butyrate, or butyrate amplified the signals elicited by binding to their receptors, or the cascade of biochemical events occurring subsequent to receptor binding. It has been reported that butyrate induced the synthesis of β-adrenergic receptors in HeLa cells (Tallman *et al.*, 1977) and their subsequent coupling to adenylate cyclase (Henneberry *et al.*, 1977), which greatly strengthens the latter hypothesis. It might explain why phentholamine plus norepinephrine induced a higher PLAP activity in butyrate treated cells. Phentholamine inhibited α-adrenergic receptors, enabling more binding of norepinephrine to β-adrenergic receptors, which were already increased in number by butyrate.

In normal human keratinocytes vitamin A exerted a prominant effect on the type of keratin synthesized (Fuchs & Green, 1981; Kim *et al.*, 1984). Therefore, enhancement of cytokeratin content (cytokeratins 5+8) induced by butyrate and not by retinoic acid was very interesting. It is tempting to assume that correlation of certain biochemical changes in the cell with cessation of cell proliferation may imply differentiation to a more mature phenotype. However, it is pertinent to note that still many important questions concerning links to cell maturation need to be answered unambiguously. It indicated that the use of alternative characterization techniques is warranted, including more research on the type of keratin expressed (Kim *et al.*, 1984; Moll *et al.*, 1982), possible detection of the precursor protein involucrin of the cornified envelope (Reiss *et al.*, 1985), or analysis of possible transglutaminase activity induced by differentiation-inducing agents (Mier *et al.*, unpublished results).

In conclusion, butyrate enhanced PLAP activity, which could be inhibited by retinoic acid or synergistically enhanced by butyrate plus catecholamines. The results obtained with antagonists showed that the effect on PLAP activity was exerted by stimulation of catecholamine-like receptors. Furthermore, butyrate increased cytokeratin content of HN-1 cells, which appeared to be due to keratins 5+8.

This work was supported by grant $G_4$/83 from the University of Nijmegen Research Pool (UOP) and in part by Ank van Vlissingen Foundation and the Maurits and Anna de Kock Foundation.

### References

ABE, M. & KUFE, D.W. (1984). Effect of sodium butyrate on human breast carcinoma (MCF-7) cellular proliferation, morphology, and CEA production. *Breast Cancer Res. Treat.*, **4**, 269.

AUGERON, C. & LABOISSE, C.L. (1984). Emergence of permanently differentiated cell clones in a human colonic cancer cell line in culture after treatment with sodium butyrate. *Cancer Res.*, **44**, 3961.

BIJMAN, J.TH., WAGENER, D.J.TH., VAN RENNES, H., WESSELS, J.M.C. & VAN DEN BROEK, P. (1985). Flow cytometric evaluation of dispersion from human head and neck tumors. *Cytometry*, **6**, 334.

BIJMAN, J.TH., WAGENER, D.J.TH., WESSELS, J.C.M., VAN DEN BROEK, P. & RAMAEKERS, F.C.S. (1986). Cell size, DNA, and cytokeratin analysis of human head and neck tumors by flow cytometry. *Cytometry*, **7**, 76.

BIJMAN, J.TH., WAGENER, D.J.TH., GRAAFSMA, S.J., WESSELS, J.M.C. & VAN DEN BROEK, P. (1987). Modulation of proliferation of a human head and neck squamous carcinoma cell line (HN-1) by catecholamines and histamine. *Anticancer Res.*, **7**, (in press).

BURKE, D.C. (1986). Interferon and cell differentiation. *Br. J. Cancer*, **53**, 301.

COWLEY, G.P., SMITH, J.A. & GUSTERSON, B.A. (1986). Increased EGF receptors on human squamous carcinoma cell lines. *Br. J. Cancer*, **53**, 223.

CRUISE, J.L., COTECCHIA, S. & MICHALOPOULES, G. (1986). Norepinephrine decreases EGF binding in primary rat hepatocyte cultures. *J. Cell. Physiol.*, **127**, 39.

EASTY, D.M., EASTY, G.C., CARTER, R.L., MONAGHAU, P. & BUTLER, L.J. (1981). Ten human carcinoma cell lines derived from squamous carcinomas from the head and neck. *Br. J. Cancer*, **43**, 772.

FUCHS, E. & GREEN, H. (1981). Regulation of terminal differentiation of cultured human keratinocytes by vitamin A. *Cell*, **25**, 617.

HENNEBERRY, R.C., SMITH, C.C. & TALLMAN, J.F. (1977). Relationships between β-adrenergic receptors and adenylate cyclase in HeLa cells. *Nature*, **268**, 252.

JETTEN, A.M. (1984). Modulation of cell growth by retinoids and their possible mechanisms of action. *Fed. Proc.*, **43**, 134.

KAMECH, N., HILL, A.M., SEIF, R. & PANTALONI, D. (1986). Butyrate converts rat 3T3 fibroblasts into giant cells. *Exp. Cell Res.*, **162**, 326.

KENNEDY, M.F.G., TUTTON, P.J.M. & BARKLA, D.H. (1985). Adrenergic factors regulating cell division in the colonic crypt epithelium during carcinogenesis and in the colonic adenoma and adenocarcinoma. *Br. J. Cancer*, **52**, 383.

KIM, K.H., SCHWARTZ, F. & FUCHS, E. (1984). Differences in keratin synthesis between normal epithelial cells and squamous cell carcinoma are mediated by vitamin A. *Proc. Natl Acad Sci. (USA)*, **81**, 4280.

KITZIS, A., TICHONICKY, L., DEFER, N. & KRUH, J. (1980). Effect of sodium butyrate on chromatin structure. *Biochem. Biophys. Res. Comm.*, **93**, 833.

MARY, P.L. & RAO, J.P. (1981). Type of adrenoceptors involved in induction by adrenaline of hepatic alkaline phosphatase. *Br. J. Pharm.*, **72**, 8.

MIER, P.D. & VAN RENNES, H. (1982*a*). Cutaneous alkaline phosphatase: a biochemical study. *Arch. Dermatol. Res.*, **274**, 221.

MIER, P.D. & VAN RENNES, H. (1982*b*). Cutaneous sialidase. *J. Invest. Dermatol.*, **78**, 267.

MOLL, R., FRANKE, W.W. & SCHILLER, D.L. (1982). The catalog of human cytokeratins: patterns of expression in normal epithelia, tumors and cultured cells. *Cell*, **31**, 11.

MORITA, A., TSAO, D. & KIM, Y.S. (1982). Effect of sodium butyrate on alkaline phosphatase in HRT-18, a human rectal cancer cell line. *Cancer Res.*, **42**, 4540.

NAKAGAWA, T., NAKAO, Y., MATSUI, T. & 4 others (1985). Effects of sodium N-butyrate on alpha-fetoprotein and albumin secretion in the human hepatoma cell oine PLC/PRF/5. *Br. J. Cancer*, **51**, 357.

PIERCE, G.B. & WALLACE, C. (1971). Differentiation of malignant to benign cells. *Cancer Res.*, **31**, 127.

PRASAD, K.N. & SINHA, P.K. (1976). Effect of sodium butyrate on mammalian cells in culture: a review. *In Vitro*, **12**, 125.

PRASAD, K.N. (1980). Butyric acid: a small fatty acid with diverse biological functions. *Life Sci.*, **27**, 1351.

RAMAEKERS, F.C.S., PUTS, J.J.G., MOESKER, O. & 6 others (1983). Antibodies to intermediate filament proteins in the immunohistochemical identification of human tumors: an overview. *Histochem. J.*, **15**, 691.

RAMAEKERS, F.C.S., BECK, H., VOOIJS, G.P. & HERMAN, C.J. (1984). Flow-cytometric analysis of mixed cell populations using intermediate filament antibodies. *Exp. Cell Res.*, **153**, 249.

REESE, D.H. & POLITANO, V.A. (1981). Evidence for the retinoid control of urothelial alkaline phosphatase. *Biochem. Biophys. Res. Comm.*, **102**, 322.

REESE, D.H., GRATZNER, H.G., BLOCK, N.L. & POLITANO, V.A. (1985). Control of growth, morphology, and alkaline phosphatase activity by butyrate and related short-chain fatty acids in the retinoid-responsive 9-1C rat prostatic adenocarcinoma cell. *Cancer Res.*, **45**, 2308.

REISS, M., PITMAN, S.W. & SARTORELLI, A.C. (1985). Modulation of the terminal differentiation of human squamous carcinoma cells *in vitro* by all-trans retinoic acid. *J. Natl Cancer Inst.*, **74**, 1015.

RIGGS, M.G., WHITTAKER, R.G., NEUMANN, J.R. & INGRAM, V.M. (1977). n-Butyrate causes histone modification in HeLa and Friend erythroleukemia cells. *Nature*, **268**, 462.

SARTORELLI, A.C. (1985). Malignant cell differentiation as a potential therapeutic approach. *Br. J. Cancer*, **52**, 293.

SCHIFF, L.J. & MOORE, S.J. (1985). Effects of cyclic adenosine 3′:5′-monophosphate elevating agents and retinoic acid on differentiation in retinoic-deficient tracheal cultures. *In Vitro Cell. Develop. Biol.*, **21**, 688.

TALLMAN, J.F., SMITH, C.C. & HENNEBERRY, R.C. (1977). Induction of functional β-adrenergic receptors in HeLa cells. *Proc. Natl Acad. Sci.* (*USA*), **74**, 873.

TUTTON, P.J.M. & BARKLA, D.H. (1980). Neural control of colonic cell proliferation. *Cancer*, **45**, 1172.